# Change in Quality of Life for Patients with Irritable Bowel Syndrome following Referral to a Gastroenterologist: A Cohort Study

**DOI:** 10.1371/journal.pone.0139389

**Published:** 2015-10-02

**Authors:** Caroline Canavan, Joe West, Timothy Card

**Affiliations:** Division of Epidemiology and Public Health, University of Nottingham, Clinical Sciences Building, City Hospital Campus, Nottingham, England, United Kingdom; University of Geneva, SWITZERLAND

## Abstract

**Background:**

Irritable bowel syndrome (IBS), a chronic functional condition, considerably reduces quality of life (QoL) and referral to gastroenterology is common. Until now, however, the impact of seeing a gastroenterologist for IBS on patients’ QoL and utility has not been assessed.

**Methods:**

Patients referred with “probable IBS” to the Nottingham Treatment Centre between October 2012 and March 2014 were invited to complete a QoL questionnaire (EuroQol–5 Dimension) before their first appointment. Patients with confirmed IBS who completed this baseline assessment were sent follow-up questionnaires three and twelve months later. Global QoL and utility were measured at each time point and change from baseline calculated. Paired t-tests analysed the significance of any change.

**Results:**

Of 205 invited patients, 69 were eligible and recruited. Response at three and twelve months was 45% and 17% respectively. Median global QoL at baseline was 67.5 (Interquartile range [IQR] 50.0 to 80.0), with a mean increase of 3.25 (95% confidence interval [CI] -5.38 to 11.88) three months later and a mean decrease of -1.82 (95% CI -16.01 to 12.38) after one year. The median utility at baseline was 0.76 (IQR 0.69 to 0.80), with a mean increase of 0.06 (95%CI -0.01 to 0.14) at three months and no change, 0.00 (-0.16 to 0.16), after one year.

**Conclusion:**

Patients experienced a small but not statistically significant increase in QoL and utility three months after seeing a gastroenterologist for IBS, which was not maintained. Gastroenterology referral does not appear to appreciably improve Qol for most people with IBS.

## Background

Irritable Bowel Syndrome (IBS) is a chronic condition characterised by abdominal pain with associated diarrhoea, constipation or both but with no structural abnormality of the bowel. IBS has no attributable mortality[1] but it is important due to the effect it has on quality of life (QoL)[2,3] and the large number of people it effects, with a global prevalence of 11%.[4]

IBS causes considerable reductions in all dimensions of QoL.[2] The effects on work, social life and ability to travel are the greatest[5,6] and most IBS patients report at least moderate pain and moderate anxiety or depression.[5–8] Symptoms fluctuate over time, but QoL does not change over three months without any intervention.[9,10] This morbidity leads to high levels of health care utilization,[9,11–16] with over half of primary care consultations for IBS being because the patient is not satisfied with their previous treatment.[13] This dissatisfaction is the principle reason primary care physicians refer patients with IBS to see a gastroenterologist.[17] Uncertainty that IBS is the correct diagnosis is the second most frequent reason.[17] This uncertainty seems most common when patients have diarrhoea predominant symptoms.[18,19] Consequently, despite guidelines recommending a positive clinical diagnosis and management in primary care,[20] around 20% of IBS patients see a gastroenterologist.[12,19] Some studies have assessed how successful seeing a gastroenterologist is for confirming the IBS diagnosis,[21–23] but none have addressed how it affects QoL, the most frequent reason for the referral.[17]

Consequently, we conducted this study to measure how consulting a gastroenterologist for IBS affects QoL. This information is essential to enable clinicians to make appropriate referral decisions and for healthcare commissioners and decision makers to optimise resource allocation.

## Methods

We screened referral letters from general practitioners to the gastroenterology outpatient clinic at the Nottingham Treatment Centre between October 2012 and March 2014 to identify patients likely to have IBS. We excluded patients with previous secondary care attendance for their symptoms mentioned in their referral letters. Potentially eligible patients with referral letters describing symptoms in keeping with a diagnosis of IBS, or who had IBS diagnosed already by the general practitioner were sent a QoL questionnaire before seeing a gastroenterologist. The questionnaire consisted of the EuroQol Questionnaire of 5-Dimensions (EQ-5D) and some supplementary questions that asked about demographics, symptoms, time off work and asked patients to confirm this was their first attendance at a gastroenterology clinic. The QoL data from this instrument is converted easily to a utility score on a scale from 0 (death) to 1 (perfect health) which is necessary for use in economic evaluation. EQ-5D has also been shown to be valid in IBS, it is sensitive to change in disease captured on disease specific instruments[5] and has good longitudinal validity.[24] If patients wished to participate, they completed the questionnaire before attending for their appointment and brought it with them. Final diagnosis of IBS was confirmed by checking participants’ medical records eight to ten weeks following their clinic appointment. Those found not to have IBS were then withdrawn from the study. Participants with confirmed IBS were sent a second questionnaire three months following their first appointment. A third questionnaire was sent to all the eligible participants one year after their initial clinic appointment, regardless of whether they returned the second questionnaire.

### Exclusion criteria

We did not invite patients aged under 16 or currently inmates in a prison to take part in the study. Patients who declined to participate, were unable to consent, had not completed the first questionnaire adequately to assess EQ-5D (including for reasons of illiteracy in written English) or did not attend the appointment were not recruited. Recruits were excluded from the study if they were diagnosed with a condition other than IBS following their clinic appointment or the clinic appointment was not their first referral for their IBS symptoms. Participants who returned invalid second or third questionnaires had that questionnaire excluded and were sent a replacement.

### Statistics

The EQ-5D health state utility value was calculated using the UK specific valuation algorithm.[25] Baseline characteristics were analysed using simple descriptive statistics. The mean difference in utility, VAS and time off work for each patient before their clinic appointment and at three and twelve months afterwards were analysed using paired t-tests with 95% confidence intervals. We stratified VAS and utility results by sex, age, whether the person had a diagnosis of IBS before they were referred and by the underlying symptoms causing the referral (pain, diarrhoea, constipation or alternating bowel habit). Mean difference from before seeing a gastroenterologist to three and twelve months afterwards was calculated with 95% confidence intervals and significance assessed using paired t-tests.

Ethics approval was granted by the East Midlands Regional Ethics Committee (code: 11/EM/0298).

## Results

**Fig 1:**
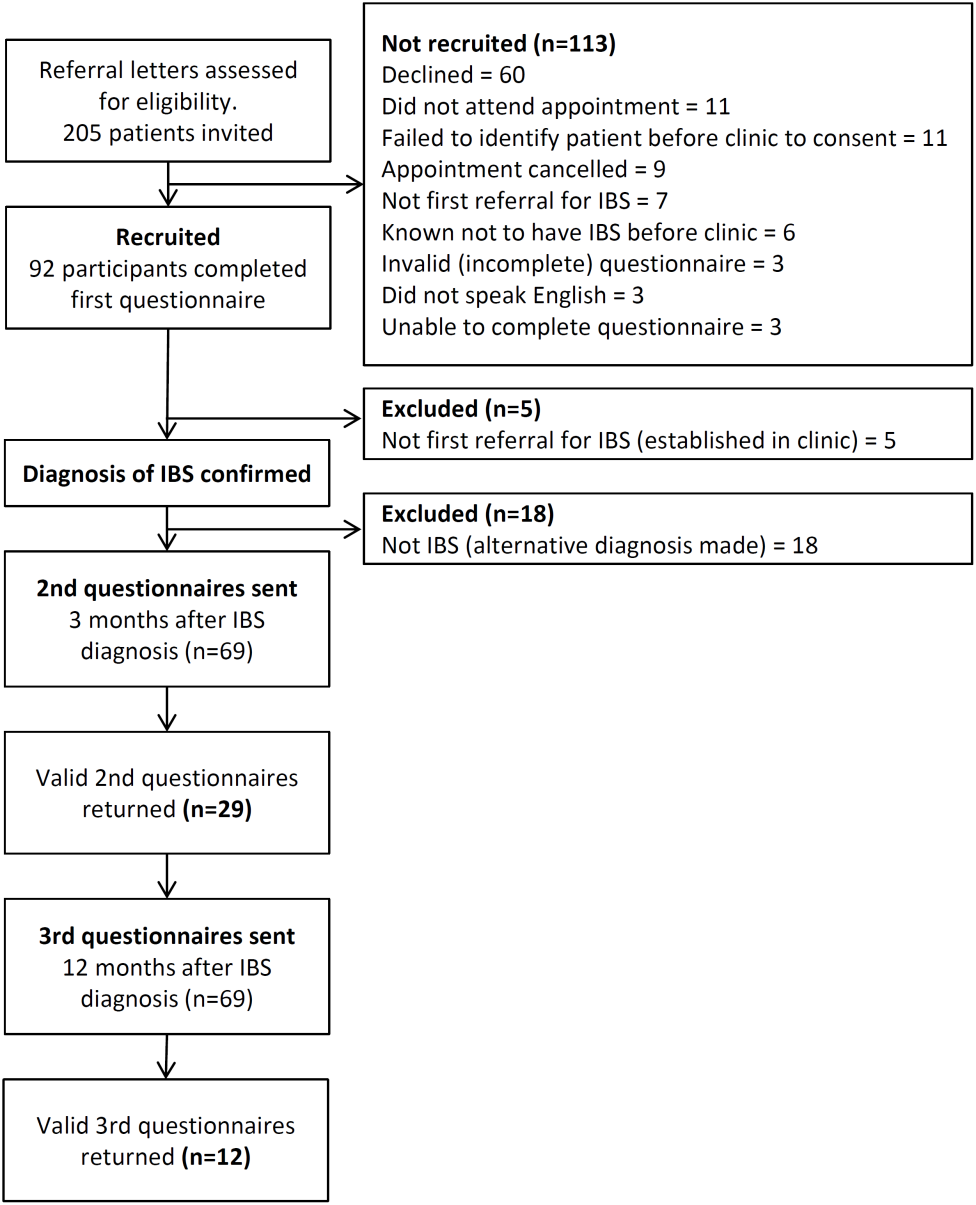
Flowchart showing the recruitment process.

We invited 205 patients to participate after screening referral letters. Of these, sixty declined, 31 did not have an initial questionnaire collected before their appointment as required in our protocol and 22 did not meet the eligibility criteria. Ninety-two patients completed the first questionnaire. Review of their notes after the appointment found that 5 had been seen previously by gastroenterologist and 18 were not diagnosed with IBS. This left 69 patients with IBS referred to see a gastroenterologist for the first time who were recruited to our study. At three months, 29 (42%) returned a follow-up questionnaire and at one year 12 (17%) returned a further questionnaire (Fig 1). Table 1 shows the demographic details of all invited patients, the initial recruits and the responders at each time point. From those invited, 41% of referrals already had a diagnosis of IBS and over half were referred with diarrhoea. Over two thirds of the patients were female, with 73% of those invited and 69% of responders being female. The mean age of all invited patients was 41.4 years (95% CI 39.3 to 43.6) and the mean age of those participating was 40.2 years (95% CI 37.2 to 43.3). The proportions of patients in each age group differed between those invited and the participant sample, with the young somewhat underrepresented amongst participants. Twenty-percent of the responders were diagnosed with a condition other than IBS following their clinic appointment and investigations (Table 2). Of these patients, 35% (8 patients) had a previous diagnosis of IBS. Referral symptoms in those diagnosed with something other than IBS were proportionate to the total sample invited (Table 1).

**Table 1:** Demographics of all patients invited to participate in this study, those who agreed to participate, those diagnosed with something other than IBS and the responders in each round who contributed the questionnaires to the analysis.

		All invited patients referred potentially with IBS	Patients who declined to participate	Responders to baseline questionnaire before GI appointment			Responders to second questionnaire 3 months after GI appointment	Responders to third questionnaire 12 months after GI appointment
				Total	Excluded	Eligible		
Total		205	113	92	23 *[18 not IBS]*	**69**	**29**	**12**
Percent of all invited		100%	55%	45%	11%	34%	14%	6%
Percent of all initial responders		-	-	100%	25% *[19*.*6% not IBS]*	75%	32%	13%
Percent of eligible participants		-	-	-	-	**100%**	**42%**	**17%**
Sex (%)	Male	55 (26.8)	25 (22.1)	30 (32.6)	9 (39.1)	**21 (30.4)**	**9 (31.0)**	**3 (25.0)**
	Female	150 (73.2)	88 (77.9)	62 (67.4)	14 (60.9)	**48 (69.6)**	**20 (69.0)**	**9 (75.0)**
Age group (%)	18–29	58 (28.3)	27 (23.9)	31 (33.7)	4 (17.4)	**27 (39.1)**	**11 (37.9)**	**5 (41.7)**
	30–49	82 (40.0)	46 (40.7)	36 (39.1)	11 (47.8)	**25 (36.2)**	**10 (34.5)**	**2 (16.6)**
	Over 50	65 (31.7)	40 (35.4)	25 (27.2)	8 (34.8)	**17 (24.6)**	**8 (27.6)**	**5 (41.7)**
Diagnosed with IBS before GI referral (%)		84 (41.0)	44 (38.9)	40 (43.5)	8 (34.8)	**32 (46.4)**	**11 (37.9)**	**6 (50.0)**
Reason for referral (%)	Abdominal pain	139 (67.8)	80 (70.8)	59 (64.1)	14 (60.9)	**43 (62.3)**	**15 (51.7)**	**10 (83.3)**
	Diarrhoea	101 (49.3)	50 (44.3)	51 (55.4)	12 (52.2)	**39 (56.5)**	**16 (55.2)**	**6 (50.0)**
	Constipation	26 (12.7)	18 (15.1)	8 (8.7)	4 (17.3)	**4 (5.8)**	**1 (3.5)**	**3 (25.0)**
	Alternating bowel habit	29 (14.2)	17 (15.0)	12 (13.0)	1 (4.3)	**11 (15.9)**	**4 (13.8)**	**1 (8.3)**

**Table 2:** Conditions diagnosed by a gastroenterologist during outpatient assessment following the first referral of patients potentially with IBS from primary care.

Condition diagnosed or confirmed by gastroenterologist		Total	% of responders
**IBS**		**74**	**80.4**
**Conditions other than IBS**		**18**	**19.6**
	Colorectal cancer	2	2.2
	Inflammatory bowel disease	2	2.2
	Obstructed defecation	2	2.2
	Coeliac disease	1	1.1
	Diverticular disease	1	1.1
	Chronic abdominal pain	1	1.1
	Gastroenteritis	1	1.1
	Faecal incontinence	1	1.1
	Rectal polyp	1	1.1
	Tropical sprue	1	1.1
	Non GI problem	5	5.4

### QoL domains

Participants reported that pain or discomfort and depression or anxiety were the two domains that contributed to the greatest loss of QoL at each time point (Table 3). Only 17% reported no problems with pain before they saw a gastroenterologist, this increased to 38% at three months, but fell again to 17% at one year. Across the three questionnaires, around 8% reported extreme pain or discomfort and around 10% reported extreme depression or anxiety. No participants reported extreme problems with mobility, self-care or activities of daily living at any time point (Table 3).

**Table 3:** EQ-5D responses by domain and median and VAS scores overall utility.

Domain of EQ-5D	Score	Baseline before gastroenterology appointment for all eligible first responders (n = 69)		Baseline before gastroenterology appointment for responders with a valid 2^nd^ or 3^rd^ questionnaire (n = 32)		3 months following gastroenterology appointment (n = 29)		12 months following gastroenterology appointment (n = 12)	
		*n*	%	*n*	%	*n*	%	*n*	%
	1	*60*	87.0	*28*	87.5	*21*	72.4	*11*	91.7
Mobility	2	*9*	13.0	*4*	12.5	*8*	27.6	*1*	8.3
	3	*0*	0.0	*0*	0.0	*0*	0.0	*0*	0.0
	1	*67*	97.1	*30*	93.8	*27*	93.1	*12*	100.0
Self-care	2	*2*	2.9	*2*	6.2	*2*	6.9	*0*	0.0
	3	*0*	0.0	*0*	0.0	*0*	0.0	*0*	0.0
	1	*43*	62.3	*17*	53.1	*17*	58.6	*10*	83.3
Activities of daily living	2	*26*	37.7	*15*	46.9	*12*	41.4	*2*	16.7
	3	*0*	0.0	*0*	0.0	*0*	0.0	*0*	0.0
	1	*12*	17.4	*7*	21.9	*11*	37.9	*2*	16.7
Pain and discomfort	2	*51*	73.9	*21*	65.6	*16*	55.2	*9*	75.0
	3	*6*	8.7	*4*	12.5	*2*	6.9	*1*	8.3
	1	*35*	50.7	*15*	46.9	*17*	60.7	*4*	33.3
Depression and anxiety	2	*26*	37.7	*14*	43.7	*8*	28.6	*7*	58.3
	3	*8*	11.6	*3*	9.4	*3*	10.7	*1*	8.3
Median overall utility score (IQR)		0.76 (0.69 to 0.80)		0.73 (0.65 to 0.78)		0.80 (0.62 to 1.00)		0.73 (0.65 to 0.76)	
*Mean overall utility score for economic analysis (95% CI)*		*0*.*68 (0*.*62 to 0*.*74)*		*0*.*68 (0*.*59 to 0*.*78)*		*0*.*73 (0*.*62 to 0*.*84)*		*0*.*69 (0*.*55 to 0*.*83)*	
Median VAS score (IQR)		67.5 (50.0 to 80.0)		65.0 (50.0 to 84.0)		75.0 (58.0 to 89.0)		60.0 (50.0 to 80.0)	

### Overall utility

**Fig 2:**
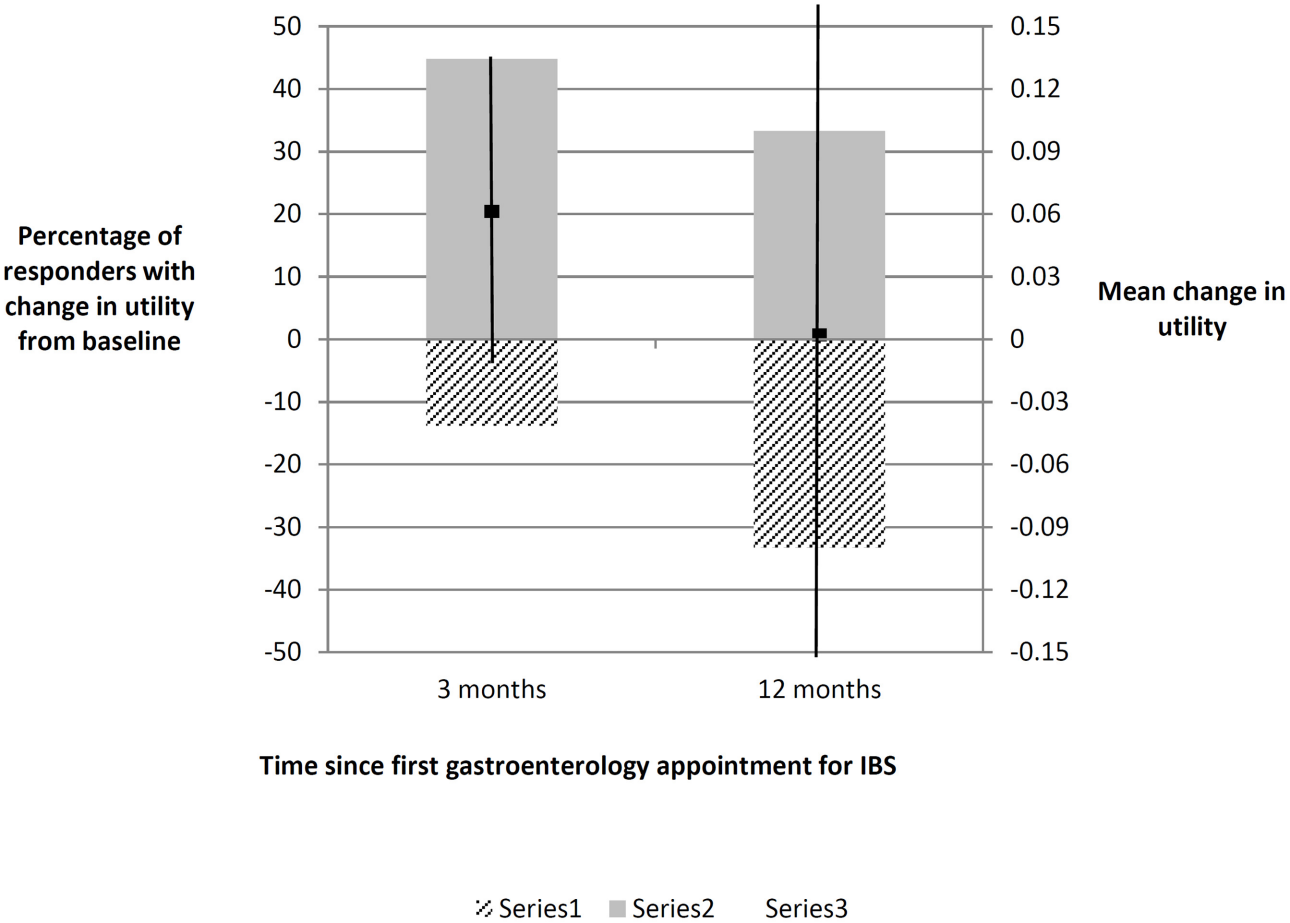
Proportion of respondents reporting a change in their utility from baseline (remaining responders reported no change) at three and twelve months with the mean utility score change and 95% confidence intervals.

The median overall utility score for the cohort before seeing a gastroenterologist was 0.76 (Interquartile range [IQR] 0.69 to 0.80). Three months after the gastroenterology appointment, 44% had improved utility, 42% experienced no change and 14% had worse utility (Fig 2). The mean utility increased by 0.04 to 0.80 (IQR 0.62 to 1.00). One year after the appointment, a third of responders had improved utility from the baseline, a third experienced no change and a third had worse utility (Fig 2). The mean utility fell by 0.07 from the 3 month peak to 0.73 (IQR 0.65 to 0.76), a 0.04 decrease from the pooled baseline. None of these changes were statistically significant, however. When responses at three and twelve months were compared to the baseline response of the same patients, as opposed to the whole cohort initially recruited, the mean utility three months after seeing a gastroenterologist had increased by 0.06 (95% CI -0.01 to 0.14), but this change reduced to 0.00 (95% CI -0.16 to 0.16) after a year (Table 4). When the results were stratified, the two groups who showed sustained increased utility were those aged over 50 years and those referred with diarrhoea. Men and women had similar utility at baseline, at three months men has greater mean improvement but this was not maintained at one year. At baseline, utility was higher in patients aged under 30. At three months those aged over 30 years reported greater mean improvement in utility which was sustained at one year, whilst those aged under 30 reported a mean decrease in utility. None of these findings were statistically significant. No changes in utility at three or twelve months were statistically significantly different from before seeing the gastroenterologist (Table 4). Mean values have been reported in Table 3 to make the results amenable to future cohort modelling.

**Table 4:** Median overall utility score at each time point stratified by demographic variables, with mean difference from baseline and the p-value from a paired t-test. Clinically significant mean changes are in **bold**. No results were statistically significant at the 5% level.

		Median before gastroenterology appointment [IQR]		Median three months afterwards [IQR]	Mean difference from baseline at 3 months [95% CI]	p-value for mean difference	Median twelve months afterwards [IQR]	Mean difference from baseline at 12 months [95% CI]	p-value for mean difference
		Patients with 3 month follow-up	Patients with 12 month follow-up						
Total		0.73 [0.62, 0.80]	0.71 [0.62, 0.80]	0.80 [0.62, 1.00]	**0.06** [-0.01, 0.14]	*0*.*10*	0.73 [0.65, 0.76]	0.00 [-0.16, 0.16]	*0*.*98*
Sex	Male	0.73 [0.62, 0.80]	0.62 [0.19, 0.62]	0.80 [0.69, 1.00]	**0.11** [-0.02, 0.23]	*0*.*10*	0.62 [0.26, 1.00]	-0.02 [-0.13, 0.08]	*0*.*42*
	Female	0.74 [0.65, 0.80]	0.76 [0.69, 0.80]	0.80 [0.62, 1.00]	**0.04** [-0.06, 0.14]	*0*.*39*	0.73 [0.73, 0.73]	0.00 [-0.22, 0.23]	*0*.*96*
Age group	18–29	0.80 [0.19, 0.88]	0.69 [0.19, 0.77]	0.69 [0.26, 0.88]	-0.01 [-0.16, 0.14]	*0*.*88*	0.73 [0.69, 0.73]	**-0.07** [-0.43, 0.30]	*0*.*64*
	30–49	0.71 [0.52, 0.80]	0.74 [0.69, 0.80]	0.82 [0.69, 1.00]	**0.13** [-0.01, 0.27]	*0*.*06*	0.73 [0.73, 0.73]	0.02 [-0.66, 0.70]	*0*.*80*
	Over 50	0.74 [0.65, 0.80]	0.76 [0.73, 0.80]	0.80 [0.67, 1.00]	**0.07** [-0.05, 0.19]	*0*.*20*	0.73 [0.62, 1.00]	**0.05** [-0.30, 0.40]	*0*.*70*
Diagnosed with IBS before GI referral		0.73 [0.52, 0.80]	0.74 [0.70, 0.80]	0.73 [0.52, 1.00]	**0.06** [-0.01, 0.13]	*0*.*07*	0.73 [0.73, 0.73]	0.01 [-0.17, 0.20]	*0*.*88*
Reason for referral	Abdominal pain	0.73 [0.19, 0.80]	0.76 [0.69, 0.80]	0.80 [0.26, 1.00]	**0.04** [-0.07, 0.15]	*0*.*48*	0.73 [0.69, 0.80]	-0.01 [-0.21, 0.20]	*0*.*94*
	Diarrhoea	0.72 [0.60, 0.80]	0.80 [0.76, 1.00]	0.74 [0.60, 0.94]	**0.03** [-0.08, 0.15]	*0*.*55*	0.73 [0.29, 0.80]	**0.12** [-0.11, 0.36]	*0*.*23*
	Constipation or Alternating	0.73 [0.62, 0.80]	0.69 [0.44, 0.74]	0.85 [0.62, 1.00]	**0.15** [-0.07, 0.37]	*0*.*13*	0.73 [0.71, 0.73]	**-0.12** [-0.56, 0.31]	*0*.*43*

### Global QoL (VAS)

Before seeing a gastroenterologist, the median VAS score for the cohort was 67.5 (IQR 50.0 to 80.0). Three months after the gastroenterology appointment, this increased to 75.0 (58.0 to 89.0), but had fallen to 60.0 (IQR 50.0 to 80.0) at one year. The mean change in VAS for individuals from before seeing a gastroenterologist to three months afterwards was not statistically significant nor was it one year later (Table 5). Table 5 shows that men experienced greater mean improvement in their global QoL three and twelve months after their gastroenterology appointment. Patients referred with diarrhoea also reported greater improvement in global QoL at both time points, whilst those referred with constipation or alternating bowel habit reported decreased global QoL after three and twelve months. None of the changes within the strata were statistically significant, however.

**Table 5:** Median overall VAS score at each time point stratified by demographic variables, with mean difference from baseline and the p-value from a paired t-test for significance of the mean change.

		Median before gastroenterology appointment [IQR]		Median three months afterwards [IQR]	Mean difference from baseline at 3 months [95% CI]	*p-value for mean difference*	Median twelve months afterwards [IQR]	Mean difference from baseline at 12 months [95% CI]	p-value for mean difference
		Patients with 3 month follow-up	Patients with 12 month follow-up						
Total		65.7 [55.0, 85.0]	62.5 [50.0, 80.0]	75.0 [58.0, 89.0]	3.25 [-5.38, 11.88]	*0*.*45*	60.0 [50.0, 80.0]	-1.82 [-16.01, 12.38]	*0*.*78*
Sex	Male	80.0 [60.0, 90.0]	60.0 [50.0, 90.0]	84.0 [75.0, 90.0]	4.33 [-10.19, 18.85]	*0*.*51*	40.0 [20.0, 60.0]	15 [-30.24, 33.26]	*0*.*65*
	Female	65.0 [50.0, 84.0]	70.0 [55.0, 80.0]	72.5 [54.0, 87.0]	2.74 [-8.90, 14.37]	*0*.*63*	60.0 [60.0, 80.0]	-1.11 [-15.46, 13.24]	*0*.*86*
Age group	18–29	70.0 [60.0, 84.0]	60.0 [25.0, 70.0]	69.0 [50.0, 85.0]	0.00 [-7.41, 7.41]	*1*.*00*	60.0 [30.0, 60.0]	-1.00 [-35.69, 33.68]	*0*.*94*
	30–49	65.0 [40.0, 86.0]	72.5 [55.0, 90.0]	81.5 [75.0, 93.0]	12.4 [-8.13, 32.93]	*0*.*21*	75.0 [70.0, 80.0]	-2.50 [-16.13, 15.63]	*0*.*87*
	Over 50	65.0 [50.0, 90.0]	80.0 [65.0, 90.0]	75.0 [55.0, 87.0]	-4.71 [-26.2, 17.09]	*0*.*62*	60.0 [55.0, 75.0]	7.50 [-18.89, 33.89]	*0*.*43*
Diagnosed with IBS before GI referral		63.0 [50.0, 90.0]	72.5 [55.0, 90.0]	79.0 [50.0, 90.0]	0.70 [-15.67, 17.06]	*0*.*93*	75.0 [60.0, 90.0]	-2.50 [-13.38, 8.38]	*0*.*58*
Reason for referral	Abdominal pain	65.5 [60.0, 80.0]	75.0 [55.0, 90.0]	69.0 [50.0, 84.0]	-1.71 [-13.53, 10.10]	*0*.*76*	60.0 [50.0, 80.0]	2.78 [-15.05, 20.61]	*0*.*73*
	Diarrhoea	63.0 [60.0, 82.0]	75.0 [65.0, 80.0]	75.0 [60.0, 87.5]	7.68 [-3.03, 18.41]	*0*.*15*	60.0 [50.0, 60.0]	15.00 [-9.83, 39.83]	*0*.*17*
	Constipation or Alternating	80.0 [50.0, 80.0]	40.0 [22.5, 72.5]	84.0 [50.0, 85.0]	-2.20 [-33.73, 29.33]	*0*.*86*	65.0 [45.0, 75.0]	-12.50 [-41.91, 16.91]	*0*.*27*

### Time off work

In the month preceding the first appointment with a gastroenterologist, the median number of days patients with IBS took off work (or away from their usual daily activities if they were not employed) for symptoms related to IBS was zero (IQR 0 to 4). Fifty-five percent of patients took no time off work due to their IBS and for those who did take time off the median time off work in a month was 4 days (IQR 2 to 14). Five patients reported that their IBS meant they were off work for the whole month. There was no statistically significant difference in the number of days patients took off work before seeing a gastroenterologist and afterwards, either at three or twelve months.

## Discussion

This is the first study to examine the effect of gastroenterology referral on QoL of patients with IBS. We found that in this group there was on average low initial quality of life, which improved somewhat 3 months after consultation, but declined to baseline after a year. We also found that in this group, who were identified as being likely to have IBS by their GPs, or from screening letters by a gastroenterology Registrar (CC), almost 20% had another organic gastrointestinal diagnosis made.

Although our study is not directly comparable to any other, and so arguably gives the best available measure of the benefit of gastroenterology referral in IBS patients, its quality needs to be examined if our results are to be correctly interpreted. We have based our work in a large teaching hospital where both sub-specialist neuro-gastroenterologists and more general luminal gastroenterologists cared for the recruited patients. To ensure that our results will be generalisable to UK secondary care referrals we therefore excluded anyone who had previously been seen in secondary care for their problem. To generate utility values alongside QoL measures and ensure that our results can be compared to others in IBS as well as other conditions, we used the generic EQ-5D questionnaire. This has been validated against the disease specific instruments for IBS[26] and the utility scores are widely used and accepted for health economic evaluations.[27]

Our study has an obvious weakness in its response rates, however, particularly at one year. This level of loss to follow up inevitably raises concerns about bias in the non-response. Though we cannot exclude the possibility of bias, when examining the known characteristics of respondents and non-respondents at each stage the groups appear similar, so such bias is at least not obvious. Despite this, it is still possible that those who are lost to follow-up have all been sufficiently reassured or had their management optimised following the gastroenterology appointment resulting in considerable improvement in QoL. If this is the case, then our study underestimates the positive impact of a referral to gastroenterology for patients with IBS. If the mean utility change for all respondents at each time point is assessed, then utility increased by 0.04 over the first three months and then decreased by 0.07 over the next nine months, so that it was 0.03 lower than baseline at one year. The magnitude of the increase in utility at three months is the same when the baseline is restricted to only those responding at three months. The magnitude of the decrease at 12 months is reduced, however. This means that the baseline utility for those patients responding at 12 months had a lower mean than those who were lost to follow-up. It is possible, therefore, that there is a response bias due to the respondents lower baseline utility representing patients who are less likely to benefit from a gastoenterology appointment. This would need further assessment in future studies. A further problem consequent upon the loss to follow up is that we have very limited power. We are therefore unable to be certain that the non-significant increase in QoL we found at 3 months is not important. In fact, the minimum clinically important difference in utility score using the EQ-5D in IBS is only 0.03,[26] so the 0.06 point mean increase which is our central estimate would clearly be important if true. Yet the results at one year suggest that even if there is a real effect on QoL three months after referral it is likely to be transient. Finally, the low power limits our ability to assess differences in subgroups of IBS patients. Notwithstanding this, the non-significant benefit we found was more pronounced in older patients with diarrhoea.

Even though there are no directly comparable studies yet published, our results should be considered in conjunction with what is already known. In keeping with other studies that report greater referral of patients with diarrhoea predominant symptoms rather than constipation or alternating bowel function[17,19,28], a higher proportion of patients in our study had diarrhoea predominant IBS than is seen in primary care.[1] Likewise, almost 20% of the 92 patients who completed a first questionnaire were diagnosed with a condition other than IBS, which is similar to a previous report in the UK.^29^ In that study, between 15% and 28% of similar patients undergoing colonoscopy had a diagnosis other than IBS apparently responsible for their symptoms.[29] These results are at variance however with those from America where a structural lesion has been reported in over 40% of patients with IBS who received a colonoscopy.[21] The proportion with such lesions, however, was not significantly different to that found in screening colonoscopy in the general population and only changed the diagnosis of IBS in 2% of patients.[21] In contrast to the higher American prevalence of apparent organic disease, in Denmark organic disease was identified in only 10% of patients diagnosed with IBS who subsequently received extensive gastroenterological investigation.[23] In the Danish study, however, patients were randomly assigned gastroenterological investigation as opposed to having been referred for further investigation as in our own.

Across Europe and North America utility values between 0.62 and 0.75 have been reported by patients with IBS[5,7,10,30] (equivalent to an average patient being willing to sacrifice between 10 and 15 years of their remaining life expectancy for an immediate cure[10]). Our baseline findings are comparable, with a median EQ-5D utility value of 0.76 (0.69 to 0.80). This is 0.10 points lower than the UK population average,[31] consistent with the reduction previously reported in IBS.[5,30] In our study, only 74% of patients had at least some pain at referral. This might seem low given that the presence of pain is required to meet the Rome III criteria. It is however slightly higher than a previous UK community based study in which only two-thirds reported some pain,[8] perhaps suggesting that complaints of pain affecting QoL increase the likelihood of referral. Pain was less common in a recent study of patients referred to a gastroenterologist with functional gastrointestinal conditions,[28] though the inclusion of conditions not requiring pain for diagnosis could clearly affect this result. Our finding that almost half experienced at least moderate depression and anxiety was the same as found in the UK community study.[8]

Despite the UK National Institute for Health and Clinical Excellence (NICE) recommending the measurement of utilities using the EQ-5D[27] for health care decision making, there are very few studies that have reported health utilities in IBS using the EQ-5D. Current guidelines are therefore based on models calculated from assumed utility changes.[20] Since these guidelines were published there have however been some studies reported of utility change following interventions in patients with IBS.[32–35] Only one of these found a change in utility which was both clinically meaningful and statistically significant, and that was from the use of a serotonin-receptor partial agonist in patients with constipation predominant IBS.[32] All the other studies like our own have been unable to demonstrate such changes,[33–35] perhaps suggesting that few if any available interventions improve global QoL or overall utility in patients with IBS.[36]

### Summary and conclusions

In an era of health austerity, it will be increasingly necessary to demonstrate the efficacy of our care. We have shown a small, non-significant, but potentially clinically meaningful mean increase in QoL for IBS patients three months after seeing a gastroenterologist. This improvement was not maintained at one year, however. Although larger studies, in particular randomised control trials, of this complex intervention may in future provide better evidence, at present the most important benefit from referral that we have been able to demonstrate may be the diagnosis of other organic pathology. Until better data are available however, the figures we provide now permit some assessment of the cost/utility of referring patients with IBS to gastroenterology services.

## Supporting Information

S1 Checklist STROBE checklist. (DOCX)

## Abbreviations

CI: Confidence interval
EQ-5D: EuroQol–5 Dimensions questionnaire
IBS: irritable bowel syndrome
IQR: Interquartile range
QoL: Quality of life
NICE: National Institute for Health and Clinical Excellence
VAS: Visual Analogue Scale
