# Supplementary material for: Change in Quality of Life for Patients with Irritable Bowel Syndrome following Referral to a Gastroenterologist: A Cohort Study
Source: PLoS One. 2015 Oct 2;10(10):e0139389. doi: 10.1371/journal.pone.0139389 (PMC4591971; doi:10.1371/journal.pone.0139389)
Supplement: S1 Checklist — (DOCX) [file pone.0139389.s001.docx]

**Document 1: STROBE Statement—checklist of items that should be included in reports of observational studies**

|  | Item No | Page of manuscript |  |
| --- | --- | --- | --- |
| **Title and abstract** | 1 | (*a*) cover page and p2 |  |
|  |  | (*b*) p2 |  |
| Introduction | | |  |
| Background/rationale | 2 | p3 |  |
| Objectives | 3 | p3 |  |
| Methods | | |  |
| Study design | 4 | p4 |  |
| Setting | 5 | p4 |  |
| Participants | 6 | (*a*) *Cohort study*—p4&5 |  |
|  |  | (*b*) *Cohort study*—no matching |  |
| Variables | 7 | p4&5 |  |
| Data sources/measurement | 8* | p4&5 |  |
| Bias | 9 | p5 |  |
| Study size | 10 | p4, figure 1 |  |
| Quantitative variables | 11 | p5 |  |
| Statistical methods | 12 | (*a*) p5 |  |
|  |  | (*b*) p5 |  |
|  |  | (*c*) p5 |  |
|  |  | (*d*) *Cohort study*—p5 |  |
|  |  | (*e*) n/a |  |
| Results | |  |  |
| Participants | 13* | (a) p6, figure1 |  |
|  |  | (b) p6, figure 1 |  |
|  |  | (c) figure 1 |  |
| Descriptive data | 14* | (a) p6 |  |
|  |  | (b) p6, table1 |  |
|  |  | (c) *Cohort study*—p6 |  |
| Outcome data | 15* | *Cohort study*—p6, table 1 |  |
| Main results | 16 | (*a*) p6-8 |  |
|  |  | (*b*) p6-8 |  |
|  |  | (*c*) n/a |  |
| Other analyses | 17 | p6-8 |  |
| Discussion | | | |
| Key results | 18 | p9 | |
| Limitations | 19 | p9-10 | |
| Interpretation | 20 | p10-11 | |
| Generalisability | 21 | p10-11 | |
| Other information | | | |
| Funding | 22 | p12 | |

*Give information separately for cases and controls in case-control studies and, if applicable, for exposed and unexposed groups in cohort and cross-sectional studies
